# Prenatal Exposure to Perfluoroalkyl Substances and Adiposity in Early and Mid-Childhood

**DOI:** 10.1289/EHP246

**Published:** 2016-06-28

**Authors:** Ana María Mora, Emily Oken, Sheryl L. Rifas-Shiman, Thomas F. Webster, Matthew W. Gillman, Antonia M. Calafat, Xiaoyun Ye, Sharon K. Sagiv

**Affiliations:** 1 Department of Environmental Health, Boston University School of Public Health, Boston, Massachusetts, USA; 2 Central American Institute for Studies on Toxic Substances, Universidad Nacional, Heredia, Costa Rica; 3 Obesity Prevention Program, Department of Population Medicine, Harvard Medical School and Harvard Pilgrim Health Care Institute, Boston, Massachusetts, USA; 4 Department of Nutrition, Harvard T.H. Chan School of Public Health, Boston, Massachusetts, USA; 5 Division of Laboratory Sciences, National Center for Environmental Health, Centers for Disease Control and Prevention, Atlanta, Georgia, USA; 6 Center for Environmental Research and Children’s Health (CERCH), School of Public Health, University of California, Berkeley, Berkeley, California, USA

## Abstract

**Background:**

Few studies have examined whether prenatal exposure to perfluoroalkyl substances (PFASs) is associated with childhood adiposity.

**Objective:**

We examined associations of prenatal exposure to PFASs with adiposity in early and mid-childhood.

**Methods:**

We measured plasma PFAS concentrations in 1,645 pregnant women (median, 9.6 weeks gestation) enrolled in Project Viva, a prospective pre-birth cohort study in Massachusetts (USA), between 1999 and 2002. We assessed overall and central adiposity in 1,006 children in early childhood (median, 3.2 years) and 876 in mid-childhood (median, 7.7 years) using anthropometric and dual X-ray absorptiometry (DXA) measurements. We fitted multivariable linear regression models to estimate exposure-outcome associations and evaluated effect modification by child sex.

**Results:**

Median (25–75th percentiles) prenatal plasma perfluorooctanoate (PFOA), perfluorooctane sulfonate (PFOS), perfluorohexane sulfonate (PFHxS), and perfluorononanoate (PFNA) concentrations in children assessed in early childhood were 5.6 (4.1–7.7), 24.8 (18.4–33.9), 2.4 (1.6–3.8), and 0.6 (0.5–0.9) ng/mL, respectively. Among girls, each interquartile range increment of prenatal PFOA concentrations was associated with 0.21 kg/m^2^ (95% CI: –0.05, 0.48) higher body mass index, 0.76 mm (95% CI: –0.17, 1.70) higher sum of subscapular and triceps skinfold thickness, and 0.17 kg/m^2^ (95% CI: –0.02, 0.36) higher DXA total fat mass index in mid-childhood. Similar associations were observed for PFOS, PFHxS, and PFNA. We observed null associations for boys and early-childhood adiposity measures.

**Conclusions:**

In this cohort, prenatal exposure to PFASs was associated with small increases in adiposity measurements in mid-childhood, but only among girls.

**Citation:**

Mora AM, Oken E, Rifas-Shiman SL, Webster TF, Gillman MW, Calafat AM, Ye X, Sagiv SK. 2017. Prenatal exposure to perfluoroalkyl substances and adiposity in early and mid-childhood. Environ Health Perspect 125:467–473; http://dx.doi.org/10.1289/EHP246

## Introduction

Perfluoroalkyl substances (PFASs) are synthetic fluorinated compounds used in the manufacture of consumer and industrial products, such as food packaging, stain-resistant coatings for carpets and furniture, insecticides, and firefighting foams (Lau et al. 2007). Human exposure to PFASs can occur through ingestion of contaminated food (Fromme et al. 2009) and drinking water (Mak et al. 2009), and ingestion or inhalation of PFAS-contaminated dust and soil (Fromme et al. 2009). Due to stable carbon-fluorine bonds, PFASs can persist in the environment and in humans (half-life of serum elimination ~ 3–5 years) (Olsen et al. 2007). For example, despite the fact that the industrial production of perfluorooctane sulfonate (PFOS) was discontinued in 2002 and reductions in perfluorooctanoate (PFOA) manufacturing emissions have been accomplished (U.S. EPA 2014), these compounds are still detectable in the U.S. general population (≥ 12 years of age) (CDC 2015).

*In vitro* and *in vivo* studies suggest that PFASs may act as endocrine disruptors via *a*) alterations in estrogen- and androgen-receptor function (Kjeldsen and Bonefeld-Jørgensen 2013), *b*) activation of peroxisome proliferator-activated receptors (PPAR) α or γ (Hines et al. 2009; Zhang et al. 2014), *c*) inhibition of the enzyme 11-β-hydroxysteroid dehydrogenase-2 (Ye et al. 2014), or *d*) disruption of thyroid hormone homeostasis (Long et al. 2013). However, the few animal and human studies that have examined the association between prenatal PFAS exposure and adiposity have reported inconsistent findings. For example, one study in mice observed higher body weights in young adult female offspring after low-dose (0.01–0.3 mg/kg) prenatal exposures to PFOA (Hines et al. 2009), whereas another rodent study reported no associations (Ngo et al. 2014). Epidemiological studies from the United States and Denmark found that prenatal PFOA serum concentrations were associated with higher body mass index (BMI), waist circumference, and body fat in 8-year-old boys and girls (Braun et al. 2016); and with overweight and high waist circumference in 20-year-old women (Halldorsson et al. 2012), but not men. Additionally, a cohort study of Greenlandic and Ukrainian children reported associations of prenatal PFOA and PFOS serum concentrations with higher risk of waist-to-height ratio > 0.5 in girls ages 5–9 years (Høyer et al. 2015). In contrast, a Danish cohort study reported that higher prenatal plasma PFOS and PFOA concentrations were associated with small decrements in BMI and waist circumference in 7-year-old boys and girls (Andersen et al. 2013). To our knowledge, none of these prospective cohort studies used direct measures of adiposity, such as dual X-ray absorptiometry (DXA), nor did they examine markers of pregnancy hemodynamics (e.g., albumin and renal function) and their potential role as confounders of the exposure-outcome association.

We examined associations of prenatal exposure to PFASs, including PFOA, PFOS, perfluorohexane sulfonate (PFHxS), and perfluorononanoate (PFNA), with adiposity in early and mid-childhood in a sample of mother–child pairs participating in Project Viva, a pre-birth cohort study in Massachusetts.

## Methods

### Study Population

Project Viva is a prospective pre-birth cohort designed to study the extent to which events during early development affect health outcomes over the lifespan. Subject recruitment and procedures for Project Viva have been described elsewhere (Oken et al. 2015; Sagiv et al. 2015). Briefly, between 1999 and 2002, we recruited pregnant women at their first prenatal visit to Atrius Harvard Vanguard Medical Associates, a multi-specialty group practice in the Greater Boston area. Eligible women were English speaking, < 22 weeks of gestation, had singleton gestations, and had no plans to move away from the study area before delivery. Of the initial 2,128 women who delivered live-born singletons, 1,668 (78%) provided a blood sample at their first prenatal visit, but only 1,645 (77%) had sufficient plasma for PFAS measurements. Of these 1,645, 1,006 (61%) children attended an in-person early childhood visit (median age, 3.2 years; range, 2.9–6.1) at the study center or at home, and 876 (53%) children attended a mid-childhood visit (median age, 7.7 years; range, 6.6–10.9). We collected body composition data from DXA in 700 (80%) of the 876 children assessed in mid-childhood.

Mothers provided written informed consent at each study visit and children provided verbal assent at the mid-childhood visit. All study activities were approved by the institutional review boards of the participating sites. The involvement of the Centers for Disease Control and Prevention (CDC) laboratory was determined not to constitute engagement in human subjects research.

### Plasma PFAS Concentrations

Plasma samples collected during the first prenatal visit (median, 9.6 weeks gestation; 25–75th percentiles, 8.7–10.9; range, 5.6–20.9) were shipped to the CDC (Atlanta, GA) for analysis. CDC staff quantified PFOA, PFOS, PFHxS, and PFNA in maternal plasma using on-line solid-phase extraction coupled with isotope dilution high-performance liquid chromatography–tandem mass spectrometry (Kato et al. 2011). Limits of detection (LOD) were 0.2 ng/mL (PFOS) and 0.1 ng/mL (all other PFASs). We detected PFASs in 99–100% of plasma and values below the LOD were replaced with the LOD divided by the square root of 2.

### Child Adiposity

We measured weight, height, hip and waist circumference, and skinfold thickness in early and mid-childhood, using procedures that have been published elsewhere (Boeke et al. 2013). Briefly, we measured height and weight using a calibrated stadiometer and scale. We calculated BMI as weight/height^2^ and computed age- and sex-specific BMI *z*-scores and percentiles using U.S. national reference data (CDC 2000). We defined obesity as BMI ≥ 95th percentile, and overweight as BMI ≥ 85th and < 95th percentile (CDC 2000), and used BMI < 85th percentile as the comparison group. We measured hip and waist circumferences using a measuring tape and computed waist-to-hip circumference ratios. We measured subscapular and triceps skinfold thicknesses using calipers and calculated the sum of the two thicknesses and the subscapular-to-triceps ratio. At age 7 years, children underwent whole body DXA scans (Discovery A Model; Hologic, Inc.) (Boeke et al. 2013). We calculated DXA fat mass, fat-free mass, and trunk fat mass indexes as mass/height^2^ (VanItallie et al. 1990).

We examined both indirect (BMI, skinfold thicknesses, and waist circumference) and direct (DXA fat mass, total fat-free mass, and trunk fat mass indexes) adiposity measures to better understand the exposure–outcome association, reduce the likelihood of outcome misclassification, and improve our ability to compare our findings to those from previous studies. We also grouped adiposity measurements into overall adiposity (BMI, BMI *z*-scores, sum of subscapular and triceps skinfold thicknesses, DXA fat mass index, and DXA total fat-free mass index) and central adiposity (waist circumference, waist-to-hip circumference ratio, subscapular-to-triceps skinfold thickness ratio, and DXA trunk fat mass index), because we were interested in exploring the associations of PFASs with body fat distribution.

### Potential Confounders and Predictors of Adiposity

Using in-person interviews and questionnaires during pregnancy, we collected information on maternal age, ethnicity, education, parity, smoking habits, marital status, dietary intake, and household income. We abstracted data on gestational weight gain (GWG), infant birth weight, and delivery date from medical records. We calculated prepregnancy BMI from self-reported height and weight. We computed GWG as the difference between prepregnancy weight and the last clinically recorded weight before delivery (median, ~ 3 days before delivery; range, 1–30) and determined gestational age at birth using the date of the last menstrual period or a second trimester ultrasound if the two estimates differed by > 10 days. We collected data on child’s physical activity, screen time, and fast food and soda intake using mailed questionnaires and in-person interviews.

### Pregnancy Hemodynamics

Plasma samples (from the same archived specimens used for quantification of PFASs) were analyzed by Children’s Hospital Boston Clinical and Epidemiologic Research Laboratory (Boston, MA) for albumin and creatinine, two markers of pregnancy hemodynamics (Savitz 2014). PFASs have high binding affinity for albumin (D’Eon et al. 2010). Furthermore, circulating albumin concentrations decrease during pregnancy, reflecting plasma volume expansion. We used plasma creatinine concentrations to compute glomerular filtration rate (GFR), a measure of the flow rate of filtered fluid through the kidney (Blackburn and Loper 1992) that increases during pregnancy and may confound the association between prenatal PFAS exposure and fetal growth (Morken et al. 2014; Verner et al. 2015). We used the Cockroft–Gault formula to compute GFR [GFR-CG = (140 – age) × pre-pregnancy weight (kg) × 1.04/plasma creatinine (μmol/L)] (Morken et al. 2014).

### Statistical Analyses

We examined associations of prenatal plasma PFAS concentrations with adiposity measurements using multivariable linear regression models. We also fitted generalized additive models (GAM) with penalized spline smooth terms for continuous exposures and visually assessed plotted splines to determine linearity of exposure–outcome associations. However, we found no evidence of nonlinear associations of prenatal PFAS concentrations with adiposity outcomes (data not shown, all *p*_GAM_ > 0.05). We therefore included PFAS concentrations parameterized as continuous variables in all models, with point estimates representing the change in outcome for each interquartile range (IQR) increase in PFAS concentrations. We interpreted our effect estimates based on their magnitude and precision instead of relying solely on their statistical significance.

We fitted multivariable polytomous logistic regression models to examine the relation of prenatal plasma PFAS concentrations with relative risks of overweight and obesity, with underweight/normal as the reference outcome. In addition, we evaluated effect modification of the exposure–outcome associations by child sex by including an interaction term in the models and running sex-stratified analyses.

Covariates that are known predictors of childhood adiposity or strong potential confounders (i.e., maternal age, ethnicity, education, parity, prepregnancy BMI, timing of blood draw, child sex, child’s age at assessment) were identified based on the existing literature (Andersen et al. 2013; Braun et al. 2016; Halldorsson et al. 2012; Høyer et al. 2015) and included *a priori* in regression models. Other potential confounders (household income, maternal smoking status, duration of breastfeeding for index child, maternal diet during pregnancy, birth weight, gestational age at birth; child’s physical activity, screen time, and fast food and soda consumption) were selected using directed acyclic graphs and then explored separately with bivariate regression models. To the models with *a priori* covariates, we added, one at a time, covariates that were associated with the exposures and any of the outcomes in the bivariate analyses (*p* < 0.10). Additional covariates (i.e., household income) were included in the final model if they materially changed the PFAS coefficients. We used chained equations to impute missing covariates (all of them missing < 10%) (Lubin et al. 2004), but excluded study participants with missing exposure or outcome data for a given exposure–outcome analysis.

We conducted several sensitivity analyses to assess the robustness of our results. First, we added all four PFASs in the final models to examine co-pollutant confounding. Second, we included maternal albumin concentrations and GFR individually to the final models to account for possible confounding by pregnancy hemodynamics. The inclusion of maternal albumin concentrations in the models could also be considered a similar procedure to the traditional adjustment for lipids for lipophilic chemicals (Abdelouahab et al. 2013), given that albumin is the major binding protein for PFASs. Because maternal PFAS concentrations have been positively associated with GWG (Ashley-Martin et al. 2016) and GWG is an important determinant of adiposity in childhood (Oken et al. 2007), we also included GWG in the final models to explore its potential effect as mediator of the PFAS–adiposity associations. Last, we fitted linear regression models with generalized estimating equations (GEEs) for repeated outcome measures to examine longitudinal relationships of PFAS concentrations with BMI, hip and waist circumference, and skinfold thickness across childhood.

## Results

### Participants’ Characteristics

Project Viva mothers were predominantly white (73%), younger than 35 years at the time of delivery (mean age ± SD = 32.5 ± 5.1 years), married or living as married (92%), multiparous (53%), and with relatively high educational attainment (71% completed college or graduate school) and household income (62% had income > $70,000/year; Table 1). Median (25–75th percentiles) prenatal plasma PFOA, PFOS, PFHxS, and PFNA concentrations in children who were assessed in early childhood were 5.6 (4.1–7.7), 24.8 (18.4–34.1), 2.4 (1.6–3.8), and 0.6 (0.5–0.9) ng/mL, respectively (Table 2). PFAS concentrations were moderately to strongly correlated with each other [(range of Spearman correlation coefficients (*r*_s_) = 0.44–0.74; see Table S1], but weakly correlated with maternal plasma GFR (*r*_s_ = –0.11–0.28) and albumin concentrations (*r*_s_ = 0.11–0.26; data not shown). Most adiposity measurements were moderately to strongly correlated with each other (*r*_s_ = 0.35–0.98, excluding waist-to-hip and subscapular-to-triceps skinfold ratios; see Table S2). Children who were assessed at early and mid-childhood had very similar distributions of exposures, covariates, and outcomes of interest (Tables 1 and 2).

**Table 1:** Characteristics of study participants (after covariate imputation) [*n* (%)].

Characteristic	Children with early childhood data (*n* = 1,006)	Children with mid-childhood data (*n* = 876)
Maternal/family characteristics
Age at enrollment (years)
< 20	31 (3)	35 (4)
20–< 35	657 (65)	565 (64)
> 35	318 (32)	276 (32)
Race/ethnicity
Black	116 (12)	134 (15)
White	737 (73)	600 (68)
Other	153 (15)	142 (16)
Married/living as married
No	76 (8)	82 (9)
Yes	930 (92)	794 (91)
Parity
0	478 (48)	421 (48)
1	367 (36)	316 (36)
≥ 2	161 (16)	139 (16)
Education
< College graduate	292 (29)	281 (32)
College graduate	383 (38)	305 (35)
Graduate school	331 (33)	290 (33)
Annual household income (US$)
< 40,000	148 (15)	155 (18)
40,000–70,000	235 (23)	191 (22)
> 70,000	623 (62)	530 (60)
Prepregnancy BMI (kg/m^2^)
Underweight (< 18.5)	27 (3)	27 (3)
Normal (18.5–24.9)	609 (61)	526 (60)
Overweight (25.0–29.9)	228 (23)	197 (22)
Obese (≥ 30)	142 (14)	126 (14)
Smoking status
Never smoked	682 (68)	620 (71)
Former smoker	211 (21)	167 (19)
Smoked during pregnancy	113 (11)	89 (10)
Breastfeeding of the index child
No	116 (12)	94 (11)
Yes	890 (88)	782 (89)
Child characteristics
Sex
Boys	531 (53)	454 (52)
Girls	475 (47)	422 (48)
BMI in early childhood (percentile)^*a*^
Underweight/normal (< 85th)	707 (72)
Overweight (85–< 95th)	188 (19)
Obese (≥ 95th)	93 (9)
BMI in mid-childhood (percentile)^*b*^
Underweight/normal (< 85th)		646 (74)
Overweight (85–< 95th)		114 (13)
Obese (≥ 95th)		111 (13)
BMI, body mass index. ^***a***^Missing data for 18 (1.8%) children in early childhood. ^***b***^Missing data for 5 (0.6%) children in mid-childhood.		

**Table 2:** Exposure and outcome distributions in the study population.

Variable	Children with early-childhood data		Children with mid-childhood data	
	*n*	Median (P25–P75)	*n*	Median (P25–P75)
Exposure
Prenatal plasma PFAS concentrations (ng/mL)
PFOS	1,006	24.8 (18.4–34.1)	876	24.7 (18.2–33.6)
PFOA	1,006	5.6 (4.1–7.7)	876	5.6 (3.9–7.6)
PFHxS	1,006	2.4 (1.6–3.8)	876	2.3 (1.6–3.7)
PFNA	1,006	0.6 (0.5–0.9)	876	0.6 (0.5–0.9)
Adiposity measurements
Early childhood
BMI (kg/m^2^)	988	16.4 (15.6–17.3)
BMI *z*-score	988	0.5 (–0.2–1.1)
Waist circumference (cm)	992	51.1 (49.2–53.4)
Waist-to-hip circumference ratio (× 100)	987	91.4 (86.8–94.7)
Sum of subscapular and triceps skinfold thickness (mm)	964	16.0 (13.8–18.8)
Subscapular-to-triceps skinfold thickness ratio (× 100)	964	62.8 (54.0–72.9)
Mid-childhood
BMI (kg/m^2^)			871	16.4 (15.4–18.2)
BMI *z*-score			871	0.4 (–0.3–1.1)
Waist circumference (cm)			873	58.3 (54.5–63.0)
Waist-to-hip circumference ratio (× 100)			861	87.2 (84.2–90.4)
Sum of subscapular and triceps skinfold thickness (mm)			873	16.6 (13.4–22.0)
Subscapular-to-triceps skinfold thickness ratio (× 100)			873	66.7 (58.1–80.6)
DXA total fat mass index (kg/m^2^)			700	3.8 (3.1–5.1)
DXA total fat-free mass index (kg/m^2^)			700	12.9 (12.1–13.9)
DXA trunk fat mass index (kg/m^2^)			700	1.2 (0.9–1.7)
Abbreviations: BMI, body mass index; DXA, dual X-ray absorptiometry; P25, 25th percentile; P75, 75th percentile; PFAS, perfluoroalkyl substance; PFHxS, perfluorohexane sulfonate; PFNA, perfluorononanoate; PFOA, perfluorooctanoate; PFOS, perfluorooctane sulfonate.				

Mother–child pairs included in the present study did not differ substantially from the initial cohort (*n* = 2,128) on most attributes, including maternal age, education, ethnicity, prepregnancy BMI, household income, and child sex and age at early or mid-childhood visits (Oken et al. 2015). Additionally, we did not observe material differences in prenatal plasma PFAS concentrations between all children who had their prenatal PFAS concentrations measured (*n* = 1,645) and children who participated in the early and/or mid-childhood assessments (data not shown).

### Prenatal PFAS and Adiposity in Early Childhood

Associations between prenatal plasma PFAS concentrations and adiposity measurements in early childhood hovered at the null in both combined (all children) and sex-stratified analyses (most *p*_int_ > 0.20; Table 3). Likewise, we did not observe consistent associations of prenatal PFAS concentrations with risk of overweight and obesity in early childhood (see Table S3).

**Table 3:** Associations of prenatal plasma PFAS concentrations with overall and central adiposity measurements in early childhood among all children and stratified by child sex [β (95% CI)].

Variable/PFAS metabolite	All children	Boys	Girls	*p*_int_
Overall adiposity
BMI (kg/m^2^)
PFOS	0.04 (0.05, 0.12)	0.02 (–0.11, 0.15)	0.04 (–0.08, 0.16)	0.56
PFOA	0.09 (–0.02, 0.19)	0.08 (–0.09, 0.26)	0.08 (–0.06, 0.21)	0.82
PFHxS	0.01 (–0.05, 0.06)	0.00 (–0.08, 0.08)	0.01 (–0.06, 0.08)	0.94
PFNA	0.02 (–0.07, 0.12)	–0.05 (–0.18, 0.08)	0.07 (–0.05, 0.20)	0.20
BMI *z*-score
PFOS	0.03 (–0.03, 0.09)	0.02 (–0.08, 0.11)	0.04 (–0.04, 0.13)	0.50
PFOA	0.05 (–0.02, 0.12)	0.03 (–0.09, 0.15)	0.06 (–0.03, 0.15)	0.62
PFHxS	0.01 (–0.03, 0.05)	0.01 (–0.05, 0.07)	0.01 (–0.04, 0.05)	0.86
PFNA	0.02 (–0.05, 0.08)	–0.03 (–0.12, 0.06)	0.05 (–0.03, 0.14)	0.22
Sum of subscapular and triceps skinfold thickness (mm)
PFOS	–0.10 (–0.36, 0.16)	–0.16 (–0.54, 0.21)	–0.04 (–0.40, 0.32)	0.44
PFOA	0.19 (–0.12, 0.49)	0.41 (–0.08, 0.90)	0.04 (–0.36, 0.44)	0.53
PFHxS	0.16 (0.01, 0.31)	0.15 (–0.09, 0.38)	0.18 (–0.03, 0.38)	0.74
PFNA	–0.01 (–0.28, 0.25)	–0.25 (–0.63, 0.13)	0.18 (–0.20, 0.55)	0.16
Central adiposity
Waist circumference (cm)
PFOS	0.05 (–0.17, 0.27)	–0.02 (–0.36, 0.31)	0.09 (–0.21, 0.38)	0.61
PFOA	0.31 (0.04, 0.57)	0.50 (0.06, 0.93)	0.14 (–0.18, 0.47)	0.24
PFHxS	0.03 (–0.10, 0.16)	0.01 (–0.20, 0.22)	0.04 (–0.13, 0.21)	0.93
PFNA	–0.01 (–0.23, 0.22)	–0.18 (–0.52, 0.15)	0.12 (–0.18, 0.42)	0.38
Waist-to-hip circumference ratio^*a*^
PFOS	0.00 (–0.35, 0.34)	–0.16 (–0.69, 0.38)	0.14 (–0.31, 0.58)	0.67
PFOA	0.18 (–0.24, 0.59)	0.49 (–0.22, 1.19)	–0.01 (–0.50, 0.49)	0.13
PFHxS	–0.01 (–0.22, 0.20)	0.09 (–0.25, 0.42)	–0.06 (–0.32, 0.19)	0.43
PFNA	0.19 (–0.17, 0.54)	0.34 (–0.20, 0.88)	0.12 (–0.34, 0.58)	0.45
Subscapular-to-triceps skinfold thickness ratio^*a*^
PFOS	0.25 (–0.70, 1.20)	0.55 (–0.76, 1.86)	–0.01 (–1.40, 1.38)	0.60
PFOA	0.55 (–0.58, 1.68)	0.15 (–1.57, 1.88)	0.88 (–0.67, 2.44)	0.45
PFHxS	–0.12 (–0.69, 0.45)	–0.34 (–1.16, 0.48)	0.03 (–0.78, 0.83)	0.43
PFNA	0.50 (–0.48, 1.47)	0.51 (–0.82, 1.84)	0.45 (–1.00, 1.91)	0.85
Abbreviations: BMI, body mass index; PFAS, perfluoroalkyl substance; PFHxS, perfluorohexane sulfonate; PFNA, perfluorononanoate; PFOA, perfluorooctanoate; PFOS, perfluorooctane sulfonate. Effect estimates represent the mean change in the outcome for a change in PFAS concentrations from the 25th to the 75th percentile (or interquartile range). Models were adjusted for maternal age, race/ethnicity, education, parity, prepregnancy BMI, timing of blood draw, household income, child sex and age at outcome assessment. Children with data on BMI and BMI *z*-score: 988 (522 boys and 466 girls); sum of subscapular and triceps skinfold thickness and subscapular-to-triceps skinfold thickness ratio: 964 (509 boys and 455 girls); waist circumference: 992 (521 boys and 471 girls); and waist-to-hip circumference ratio: 987 (519 boys and 468 girls). ^***a***^Ratios were multiplied by 100 to make estimates interpretable.				

### Prenatal PFAS and Adiposity in Mid-Childhood

We did not find strong associations between prenatal plasma PFAS concentrations and adiposity in mid-childhood in analyses of boys and girls combined (Table 4). When we stratified by child sex, we observed null PFAS–adiposity associations among boys. However, among girls, we found small and consistent associations of prenatal PFAS concentrations with higher overall and central adiposity measures (Table 4). For example, among girls, higher PFOA concentrations were associated with modest increases in BMI [β per IQR increase = 0.21 kg/m^2^; 95% confidence interval (CI): –0.05, 0.48; *p*_int_ = 0.20], sum of subscapular and triceps skinfold thickness (β = 0.76 mm; 95% CI: –0.17, 1.70; *p*_int_ = 0.30), DXA total fat mass index (β = 0.17 kg/m^2^; 95% CI: –0.02, 0.36; *p*_int_ = 0.31), waist circumference (β = 0.46 cm; 95% CI: –0.29, 1.20; *p*_int_ = 0.19), and subscapular-to-triceps skinfold thickness ratio (β = 1.66; 95% CI: –0.16, 3.47; *p*_int_ = 0.08; see Figure S1). Similar findings were observed for PFOS, PFHxS, and PFNA (Table 4).

**Table 4:** Associations of prenatal plasma PFAS concentrations with overall and central adiposity measurements in mid-childhood among all children and stratified by child sex [β (95% CI)].

PFAS/metabolite	All children	Boys	Girls	*p*_int_
Overall adiposity
BMI (kg/m^2^)
PFOS	0.16 (–0.04, 0.36)	–0.02 (–0.32, 0.28)	0.28 (0.02, 0.55)	0.22
PFOA	0.13 (–0.10, 0.35)	–0.06 (–0.45, 0.33)	0.21 (–0.05, 0.48)	0.20
PFHxS	0.04 (–0.08, 0.17)	–0.07 (–0.27, 0.13)	0.11 (–0.05, 0.26)	0.15
PFNA	0.17 (–0.03, 0.36)	0.00 (–0.30, 0.30)	0.30 (0.06, 0.55)	0.31
BMI *z*-score
PFOS	0.07 (0.00, 0.13)	0.03 (–0.06, 0.13)	0.09 (–0.01, 0.18)	0.48
PFOA	0.04 (–0.03, 0.12)	–0.04 (–0.17, 0.08)	0.09 (–0.01, 0.18)	0.06
PFHxS	0.01 (–0.03, 0.05)	–0.01 (–0.07, 0.06)	0.01 (–0.04, 0.07)	0.52
PFNA	0.04 (–0.02, 0.11)	0.02 (–0.08, 0.11)	0.07 (–0.02, 0.16)	0.67
Sum of subscapular and triceps skinfold thickness (mm)
PFOS	0.47 (–0.16, 1.10)	0.07 (–0.80, 0.95)	0.75 (–0.18, 1.67)	0.37
PFOA	0.49 (–0.23, 1.20)	–0.11 (–1.25, 1.04)	0.76 (–0.17, 1.70)	0.30
PFHxS	0.25 (–0.14, 0.64)	–0.23 (–0.81, 0.36)	0.57 (0.03, 1.10)	0.06
PFNA	0.62 (0.01, 1.22)	0.13 (–0.74, 1.01)	1.01 (0.16, 1.86)	0.41
DXA total fat mass index (kg/m^2^)
PFOS	0.11 (–0.03, 0.25)	0.02 (–0.18, 0.22)	0.18 (–0.02, 0.38)	0.36
PFOA	0.13 (–0.02, 0.29)	0.05 (–0.22, 0.33)	0.17 (–0.02, 0.36)	0.31
PFHxS	0.04 (–0.04, 0.13)	–0.04 (–0.18, 0.09)	0.09 (–0.02, 0.19)	0.09
PFNA	0.08 (–0.07, 0.23)	–0.04 (–0.23, 0.16)	0.21 (–0.02, 0.44)	0.28
DXA total fat-free mass index (kg/m^2^)
PFOS	0.08 (–0.01, 0.18)	0.05 (–0.09, 0.19)	0.11 (–0.03, 0.24)	0.63
PFOA	0.06 (–0.05, 0.17)	0.04 (–0.15, 0.23)	0.07 (–0.06, 0.20)	0.60
PFHxS	0.00 (–0.05, 0.06)	–0.02 (–0.11, 0.07)	0.01 (–0.06, 0.09)	0.52
PFNA	0.08 (–0.02, 0.18)	0.09 (–0.05, 0.22)	0.05 (–0.11, 0.21)	0.75
Central adiposity
Waist circumference (cm)
PFOS	0.34 (–0.19, 0.87)	–0.02 (–0.78, 0.74)	0.60 (–0.15, 1.34)	0.32
PFOA	0.20 (–0.39, 0.80)	–0.33 (–1.32, 0.66)	0.46 (–0.29, 1.20)	0.19
PFHxS	0.11 (–0.22, 0.43)	–0.15 (–0.66, 0.36)	0.24 (–0.18, 0.67)	0.23
PFNA	0.31 (–0.19, 0.82)	0.11 (–0.66, 0.87)	0.50 (–0.17, 1.18)	0.79
Waist-to-hip circumference ratio^*a*^
PFOS	0.38 (–0.04, 0.80)	0.35 (–0.32, 1.03)	0.36 (–0.14, 0.86)	0.69
PFOA	0.11 (–0.36, 0.59)	0.02 (–0.86, 0.90)	0.19 (–0.32, 0.69)	0.79
PFHxS	0.19 (–0.07, 0.45)	0.06 (–0.40, 0.52)	0.24 (–0.04, 0.53)	0.74
PFNA	0.37 (–0.03, 0.77)	0.42 (–0.25, 1.10)	0.34 (–0.12, 0.80)	0.66
Subscapular-to-triceps skinfold thickness ratio^*a*^
PFOS	0.96 (–0.31, 2.23)	0.43 (–1.37, 2.24)	1.30 (–0.50, 3.10)	0.41
PFOA	1.02 (–0.41, 2.45)	–0.35 (–2.71, 2.00)	1.66 (–0.16, 3.47)	0.08
PFHxS	0.74 (–0.04, 1.52)	–0.50 (–1.70, 0.71)	1.61 (0.58, 2.65)	< 0.01
PFNA	1.78 (0.57, 2.98)	1.23 (–0.58, 3.03)	2.17 (0.52, 3.83)	0.44
DXA trunk fat mass index (kg/m^2^)
PFOS	0.05 (–0.02, 0.11)	0.02 (–0.07, 0.10)	0.07 (–0.02, 0.16)	0.53
PFOA	0.06 (–0.01, 0.13)	0.05 (–0.07, 0.17)	0.07 (–0.01, 0.16)	0.55
PFHxS	0.02 (–0.02, 0.06)	–0.02 (–0.07, 0.04)	0.04 (–0.01, 0.09)	0.12
PFNA	0.04 (–0.03, 0.11)	0.01 (–0.08, 0.09)	0.07 (–0.03, 0.18)	0.62
Abbreviations: BMI, body mass index; DXA, dual X-ray absorptiometry; PFAS, perfluoroalkyl substance; PFHxS, perfluorohexane sulfonate; PFNA, perfluorononanoate; PFOA, perfluorooctanoate; PFOS, perfluorooctane sulfonate. Effect estimates represent the mean change in the outcome for a change in PFAS concentrations from the 25th to the 75th percentile (or interquartile range). Models were adjusted for maternal age, race/ethnicity, education, parity, prepregnancy BMI, timing of blood draw, household income, child sex and age at outcome assessment. Children with data on BMI and BMI *z*-score: 871 (450 boys and 421 girls); sum of subscapular and triceps skinfold thickness and subscapular-to-triceps skinfold thickness ratio: 873 (451 boys and 422 girls); DXA total fat mass, total fat-free mass, and trunk fat mass indexes: 700 (356 boys and 344 girls); waist circumference: 873 (453 boys and 420 girls); and waist-to-hip circumference ratio: 861 (448 boys and 413 girls). ^***a***^Ratios were multiplied by 100 to make estimates interpretable.				

We observed that, among girls, those with higher prenatal PFAS concentrations were relatively more likely to be obese at 7 years (see Table S3). For example, IQR increases in prenatal PFHxS were associated with relative risks of 1.14 (95% CI: 0.97, 1.33; *p*_int_ = 0.22) for obesity. Associations for PFASs and relative risk of obesity among boys hovered at the null.

### Sensitivity Analyses

When we adjusted for all four PFASs in the same model, we observed mostly null PFAS–adiposity associations in early and mid-childhood (see Tables S4 and S5), possibly due to the moderate-to-strong correlation between PFASs and loss of precision. The inclusion of maternal plasma albumin concentrations during pregnancy (see Tables S6 and S7) and GWG (data not shown) in the models also attenuated most of the effect estimates, whereas including maternal GFR during pregnancy in the adjusted models marginally strengthened the associations (see Tables S6 and S7). We did not find consistent associations for prenatal PFAS plasma concentrations with repeated adiposity measurements in GEE analyses (see Table S8).

## Discussion

We did not find strong associations between prenatal plasma PFAS concentrations and adiposity in children from Project Viva. We did observe modest associations of prenatal exposure to PFAS with overall and central adiposity measurements and risk of obesity in girls, but not boys, in mid-childhood. Notably, we found null associations between prenatal PFAS concentrations and adiposity earlier in childhood.

To our knowledge, this is one of the largest and most comprehensive studies to date on the potential obesogenic effects of prenatal exposure to PFASs. Few studies have prospectively examined the association of prenatal exposure to PFASs with adiposity measurements (Andersen et al. 2013; Braun et al. 2016; Halldorsson et al. 2012; Høyer et al. 2015), and there are some consistencies between their findings and ours, particularly stronger associations among females. A Danish study (*n* = 665) observed positive associations of prenatal PFOA serum concentrations with BMI and waist circumference in 20-year-old women (Halldorsson et al. 2012), but not in men of the same age. Similarly, a study of Greenlandic and Ukrainian children 5–9 years of age (*n* = 1,022) found that higher prenatal PFOA and PFOS serum concentrations were associated with an increased risk of having a waist-to-height ratio > 0.5 (indirect measure of central adiposity) during childhood, particularly among girls (Høyer et al. 2015). Other studies were less consistent, however. A prospective study of U.S. children 8 years of age (*n* = 204) observed nonlinear associations of prenatal PFOA serum concentration with higher adiposity measurements in both boys and girls (Braun et al. 2016). In addition, a Danish study (*n* = 811) found that higher prenatal plasma PFOS and PFOA concentrations were associated with slightly lower BMIs and waist circumferences in both boys and girls at age 7 years (Andersen et al. 2013). Inconsistent findings could be attributable to differences in sociodemographic characteristics of the study populations, routes of exposure, maternal PFAS concentrations, outcome definitions, or analytical approaches.

In the present study, prenatal plasma PFAS concentrations showed small but positive associations with both overall and central adiposity measurements in mid-childhood among girls. Central adiposity reflects intra-abdominal fat, which is more metabolically active than fat stored in other regions of the body (Björntorp 1991). Nevertheless, recent studies have observed that overall and central adiposity measurements are similarly associated with cardiovascular disease events in adults (Taylor et al. 2010) and children (Lawlor et al. 2010).

Hemodynamic changes during pregnancy may affect circulating plasma concentrations of PFASs (Fei et al. 2007; Verner et al. 2015) and also predict unfavorable health outcomes among offspring (Savitz 2007). In our analyses, we assessed two markers of pregnancy hemodynamics, GFR and albumin, which have been examined in only two epidemiological studies of PFAS exposure (Verner et al. 2015; Whitworth et al. 2012); both studies analyzed birth weight as their outcome of interest. When we included maternal GFR in the adjusted models, we observed a subtle strengthening of the effect estimates. It is possible that, because maternal plasma samples were collected early in pregnancy, changes in GFR may have not yet influenced PFAS significantly (Verner et al. 2015). Another possibility is that GFR does not confound associations with environmental chemicals (Vesterinen et al. 2015) or that GFR confounds the associations with outcomes at birth but not at mid-childhood. When we adjusted for maternal albumin concentrations during pregnancy in our models, we noticed a slight attenuation of the effect estimates for all PFASs. A similar finding to the latter was observed in a Norwegian birth cohort study (*n* = 901) that examined the association between prenatal plasma PFOS concentrations and birth weight (Whitworth et al. 2012). These authors suggested that the estimates’ attenuation could be attributable to the binding affinity of PFAS for albumin and the association of maternal albumin concentrations with adverse pregnancy outcomes, such as preterm birth (Whitworth et al. 2012).

Findings from our analyses and previous animal (Hines et al. 2009) and human (Halldorsson et al. 2012; Høyer et al. 2015) studies suggest that prenatal exposure to PFASs may affect males and females differently. However, we can only speculate as to what the biological basis for these sex differences is. Growing evidence suggests that the association of early-life exposure to certain environmental toxicants with placental functions and risk of disease later in life may vary by child sex (Gabory et al. 2013). Consequently, one explanation could be that sex differences in the association between prenatal PFAS exposure and childhood adiposity are mediated by increased cortisol levels (Zhao et al. 2011) and linked to sex-specific effects on placental epigenetic processes, as has been reported with maternal stress (Mina et al. 2015).

Our study has several limitations. First, as in any cohort study, there was considerable loss to follow-up during the study period, which would have resulted in bias if attrition had been related to both PFAS exposure and adiposity; yet we did not find differences in PFAS concentrations between children who had their prenatal PFAS concentrations measured and those who participated in the early and/or mid-childhood assessments. Second, we cannot rule out the possibility that some associations were due to chance. Nevertheless, given that conventional approaches for correcting for multiple comparisons have low efficiency and poor accuracy (Rothman et al. 2008), we were careful to point out patterns in our results rather than highlight only isolated findings. Third, because PFASs were moderately to strongly correlated in our study population, the inclusion of all four PFASs in the final models compromised the precision and plausibility of the effect estimates and limited our ability to assess co-pollutant confounding. Fourth, the extent to which our results are generalizable to other populations may be limited because Project Viva participants had health insurance and were, on average, of higher socioeconomic status. Last, we cannot exclude the possibility that there is residual confounding by diet and that adiposity in early and mid-childhood could be also influenced by postnatal exposure to PFASs.

The limitations of the present study are offset by notable strengths, including its large sample size and longitudinal design. We measured plasma PFAS concentrations early in pregnancy and evaluated children in early and mid-childhood using detailed outcome assessments, including DXA measurements, which provide accurate results for total body composition and fat content and reduce the likelihood of outcome misclassification. In addition, we were also able to examine and/or adjust for several important prenatal and postnatal factors such as markers of pregnancy hemodynamics, maternal education, prepregnancy BMI, and household income.

## Conclusion

Our findings do not indicate a strong association between prenatal PFAS exposure and adiposity in early and mid-childhood, but rather modest associations with overall and central adiposity measurements and risk of obesity in mid-childhood among girls only. Given the ubiquity of PFASs in the environment and in human biological samples, it is of considerable public health importance to continue examining the endocrine-disrupting properties of these chemicals, even if only small effects are observed.

## Supplemental Material

(284 KB) PDF
