# Supplementary material for: Prenatal Exposure to Perfluoroalkyl Substances and Adiposity in Early and Mid-Childhood
Source: Environ Health Perspect. 2016 Jun 28;125(3):467–73. doi: 10.1289/EHP246 (PMC5332178; doi:10.1289/EHP246)
Supplement: (284 KB) PDF [file EHP246.s001.acco.pdf]

*Environ Health Perspect*
DOI: 10.1289/EHP246

**Note to readers with disabilities:** *EHP* strives to ensure that all journal content is accessible to all readers. However, some figures and Supplemental Material published in *EHP* articles may not conform to 508 standards due to the complexity of the information being presented. If you need assistance accessing journal content, please contact ehp508@niehs.nih.gov. Our staff will work with you to assess and meet your accessibility needs within 3 working days.

# Supplemental Material

# Prenatal Exposure to Perfluoroalkyl Substances and Adiposity in Early and Mid-Childhood

Ana María Mora, Emily Oken, Sheryl L. Rifas-Shiman, Thomas F. Webster, Matthew W. Gillman, Antonia M. Calafat, Xiaoyun Ye, and Sharon K. Sagiv

**Table of Contents**

| **Supplemental Table/Figure** |
|---|
| Table S1. Spearman correlation coefficients for prenatal plasma PFAS concentrations in children who had an early childhood assessment. |
| Table S2. Spearman correlation coefficients for adiposity measurements collected in mid-childhood. |
| Table S3. Associations of prenatal plasma PFAS concentrations with overweight and obesity in early and mid-childhood among all children and stratified by child sex. |
| Figure S1. Mean change (95% CI) in mid-childhood adiposity measurements associated with an IQR increment in prenatal plasma PFOA concentrations. |
| Table S4. Associations of prenatal plasma PFAS concentrations with overall and central adiposity measurements in early childhood among all children and stratified by child sex (models adjusted for all PFAS simultaneously). |
| Table S5. Associations of prenatal plasma PFAS concentrations with overall and central adiposity measurements in mid-childhood among all children and stratified by child sex (models adjusted for all PFAS simultaneously). |
| Table S6. Associations of prenatal plasma PFAS concentrations with overall and central |

adiposity measurements in early childhood among all children and stratified by child sex, after adjusting for maternal GFR and albumin levels during pregnancy.

Table S7. Associations of prenatal plasma PFAS concentrations with overall and central adiposity measurements in mid-childhood among all children and stratified by child sex, after adjusting for maternal GFR and albumin levels during pregnancy.

Table S8. Longitudinal associations of prenatal plasma PFAS concentrations with overall and central adiposity measurements collected in early and mid-childhood, estimated using linear regression models with generalized estimation equations (GEE).

**Table S1.** Spearman correlation coefficients for prenatal plasma PFAS concentrations in children who had an early childhood assessment (*n* = 1,006).

| **Chemical** | **PFOS** | **PFOA** | **PFHxS** | **PFNA** |
|---|---|---|---|---|
| PFOS | 1.00 | | | |
| PFOA | 0.74 | 1.00 | | |
| PFHxS | 0.50 | 0.53 | 1.00 | |
| PFNA | 0.62 | 0.55 | 0.44 | 1.00 |

PFAS, perfluoroalkyl substances; PFHxS, perfluorohexane sulfonate; PFNA, perfluorononanoate; PFOA, perfluorooctanoate; PFOS, perfluorooctane sulfonate.

*All coefficients significant at a p-value of <0.01.

**Table S2.** Spearman correlation coefficients for adiposity measurements collected in mid-childhood (*n* = 693).

| Measurement | *Overall adiposity* | | | | | *Central adiposity* | | | |
|---|---|---|---|---|---|---|---|---|---|
| | BMI ($kg/m^2$) | BMI z-score | Sum of subscapular and triceps skinfold thickness (mm) | DXA total fat mass index ($kg/m^2$) | DXA total fat-free mass index ($kg/m^2$) | Waist circumference (cm) | Waist-to-hip circumference ratio | Subscapular-to-triceps skinfold thickness ratio | DXA trunk fat mass index ($kg/m^2$) |
| ***Overall adiposity*** | | | | | | | | | |
| BMI ($kg/m^2$) | 1.00 | | | | | | | | |
| BMI z-score | 0.98 | 1.00 | | | | | | | |
| Sum of subscapular and triceps skinfold thickness (mm) | 0.80 | 0.78 | 1.00 | | | | | | |
| DXA total fat mass index ($kg/m^2$) | 0.81 | 0.80 | 0.90 | 1.00 | | | | | |
| DXA total fat-free mass index ($kg/m^2$) | 0.78 | 0.77 | 0.41 | 0.37 | 1.00 | | | | |
| ***Central adiposity*** | | | | | | | | | |
| Waist circumference (cm) | 0.86 | 0.83 | 0.73 | 0.73 | 0.66 | 1.00 | | | |
| Waist-to-hip circumference ratio | 0.26 | 0.28 | 0.19 | 0.23 | 0.20 | 0.50 | 1.00 | | |
| Subscapular-to-triceps skinfold thickness ratio | 0.20 | 0.19 | 0.12 | 0.10 | 0.23 | 0.21 | 0.18 | 1.00 | |
| DXA trunk fat mass index ($kg/m^2$) | 0.78 | 0.77 | 0.89 | 0.98 | 0.35 | 0.72 | 0.25 | 0.13 | 1.00 |

BMI, body mass index; DXA, dual X-ray absorptiometry.
*All coefficients significant at a p-value of <0.01.

**Table S3.** Associations of prenatal plasma PFAS concentrations with overweight and obesity in early and mid-childhood among all children and stratified by child sex.[a,b,c]

| | **All children** | **Boys** | **Girls** | $p_{int}$ |
|---|---|---|---|---|
| | **RRR (95% CI)** | **RRR (95% CI)** | **RRR (95% CI)** | |
| ***Early childhood*** | | | | |
| Overweight (85th-<95th vs. <85th) | | | | |
| PFOS | 1.07 (0.92, 1.24) | 1.04 (0.82, 1.31) | 1.11 (0.90, 1.36) | 0.36 |
| PFOA | 1.05 (0.87, 1.26) | 0.96 (0.70, 1.31) | 1.10 (0.88, 1.38) | 0.33 |
| PFHxS | 1.03 (0.94, 1.13) | 1.02 (0.88, 1.17) | 1.05 (0.93, 1.18) | 0.73 |
| PFNA | 1.12 (0.96, 1.30) | 1.03 (0.81, 1.30) | 1.20 (0.97, 1.49) | 0.24 |
| Obese (≥95th vs. <85th) | | | | |
| PFOS | 0.97 (0.76, 1.23) | 0.95 (0.68, 1.32) | 0.96 (0.65, 1.43) | 0.75 |
| PFOA | 1.03 (0.80, 1.32) | 0.98 (0.67, 1.45) | 1.05 (0.72, 1.52) | 0.75 |
| PFHxS | 1.02 (0.89, 1.17) | 0.94 (0.75, 1.17) | 1.07 (0.90, 1.26) | 0.43 |
| PFNA | 0.97 (0.75, 1.27) | 0.85 (0.59, 1.23) | 1.03 (0.69, 1.55) | 0.46 |
| ***Mid-childhood*** | | | | |
| Overweight (85th-<95th vs. <85th) | | | | |
| PFOS | 1.15 (0.95, 1.40) | 1.20 (0.92, 1.57) | 1.08 (0.80, 1.46) | 0.63 |
| PFOA | 1.02 (0.80, 1.29) | 0.94 (0.64, 1.40) | 1.06 (0.78, 1.44) | 0.36 |
| PFHxS | 1.04 (0.92, 1.17) | 0.95 (0.77, 1.19) | 1.11 (0.95, 1.29) | 0.16 |
| PFNA | 1.06 (0.85, 1.32) | 1.11 (0.83, 1.48) | 0.99 (0.67, 1.46) | 0.64 |
| Obese (≥95th vs. <85th) | | | | |
| PFOS | 1.12 (0.90, 1.38) | 1.02 (0.73, 1.42) | 1.20 (0.90, 1.60) | 0.86 |
| PFOA | 1.10 (0.88, 1.37) | 0.97 (0.64, 1.48) | 1.15 (0.88, 1.51) | 0.73 |
| PFHxS | 1.07 (0.94, 1.22) | 0.91 (0.70, 1.18) | 1.14 (0.97, 1.33) | 0.22 |
| PFNA | 1.21 (0.99, 1.47) | 1.12 (0.78, 1.60) | 1.31 (1.00, 1.70) | 0.81 |

PFAS, perfluoroalkyl substances; PFHxS, perfluorohexane sulfonate; PFNA, perfluorononanoate; PFOA, perfluorooctanoate; PFOS, perfluorooctane sulfonate.

[a]Effect estimates represent the risk of overweight (BMI ≥85th and <95th percentile) or obesity (BMI ≥95th percentile) relative to underweight/normal weight (BMI <85th percentile) for a change in PFAS concentrations from the 25th to the 75th percentile (or interquartile range).

[b]Models adjusted for maternal age, race/ethnicity, education, parity, pre-pregnancy BMI, timing of blood draw, household income, and child sex, child's age at outcome assessment.

[c]Children at early childhood visit = overweight: 188 (103 boys and 85 girls) and obese: 93 (59 boys and 34 girls); mid-childhood visit = overweight: 114 (62 boys and 52 girls) and obese: 111 (54 boys and 57 girls).

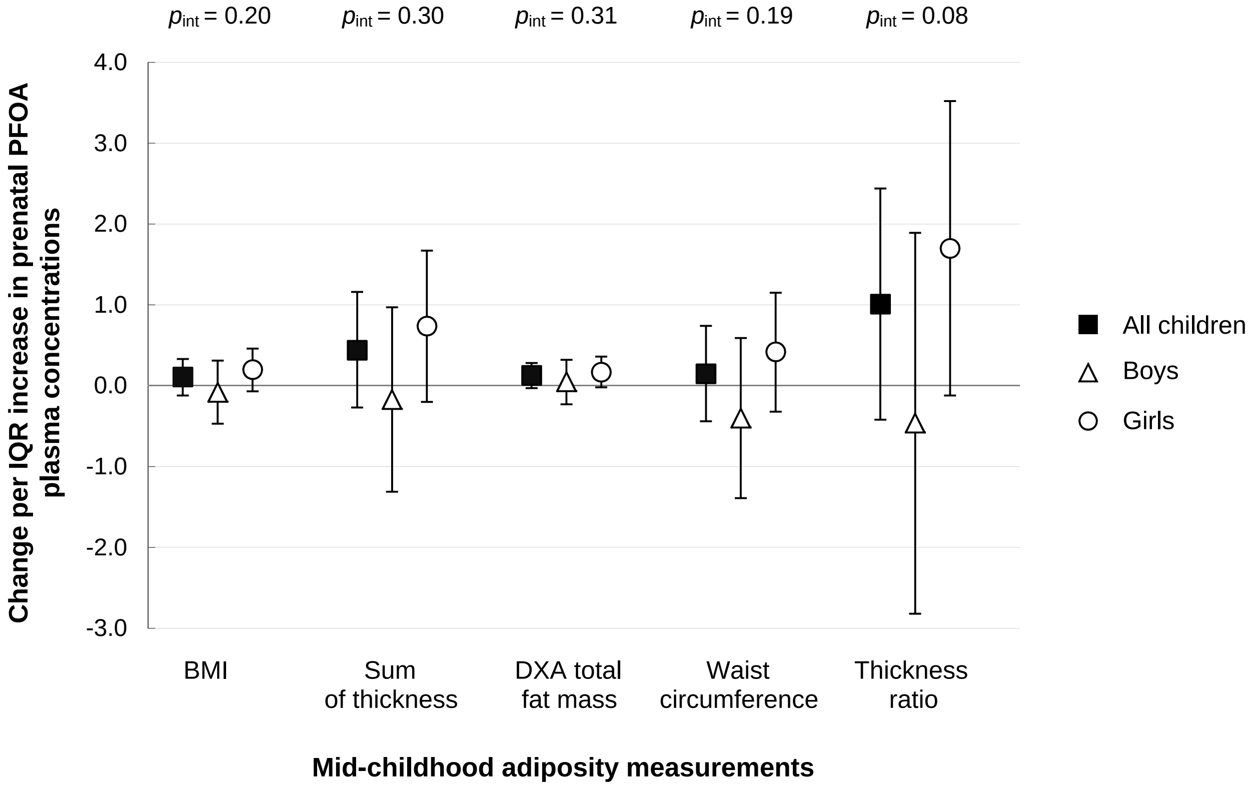


**Figure S1.** Mean change (+ 95% confidence intervals) in mid-childhood adiposity measurements associated with an IQR increment in prenatal plasma PFOA concentrations. Models adjusted for maternal age, race/ethnicity, education, parity, pre-pregnancy BMI, timing of blood draw, household income, child sex and age at outcome assessment. Subscapular-to-triceps skinfold thickness ratio was multiplied by 100 to make estimate interpretable. *Abbreviations*: BMI, body mass index; DXA, dual X-ray absorptiometry, IQR, interquartile range; PFOA, perfluorooctanoate.

**Table S4.** Associations of prenatal plasma PFAS concentrations with overall and central adiposity measurements in early childhood among all children and stratified by child sex (models adjusted for all PFAS simultaneously).[a,b,c]

| | All children | Boys | Girls |
|---|---|---|---|
| | **β (95% CI)** | **β (95% CI)** | **β (95% CI)** |
| ***Overall adiposity*** | | | |
| BMI (kg/m$^2$) | | | |
| PFOS | -0.02 (-0.14, 0.11) | 0.00 (-0.17, 0.18) | -0.03 (-0.21, 0.14) |
| PFOA | 0.10 (-0.04, 0.24) | 0.14 (-0.09, 0.37) | 0.08 (-0.11, 0.26) |
| PFHxS | 0.00 (-0.06, 0.05) | -0.01 (-0.10, 0.08) | 0.00 (-0.07, 0.08) |
| PFNA | 0.00 (-0.10, 0.10) | -0.10 (-0.25, 0.06) | 0.06 (-0.08, 0.21) |
| BMI z-score | | | |
| PFOS | 0.01 (-0.08, 0.09) | 0.02 (-0.10, 0.14) | 0.00 (-0.12, 0.12) |
| PFOA | 0.04 (-0.05, 0.14) | 0.04 (-0.12, 0.20) | 0.04 (-0.08, 0.17) |
| PFHxS | 0.00 (-0.04, 0.04) | 0.01 (-0.05, 0.07) | 0.00 (-0.05, 0.05) |
| PFNA | 0.00 (-0.08, 0.07) | -0.06 (-0.17, 0.05) | 0.03 (-0.06, 0.13) |
| Sum of subscapular and triceps skinfold thickness (mm) | | | |
| PFOS | -0.41 (-0.77, -0.05) | -0.48 (-0.97, 0.01) | -0.31 (-0.83, 0.21) |
| PFOA | 0.42 (0.02, 0.83) | 0.95 (0.29, 1.60) | 0.11 (-0.44, 0.65) |
| PFHxS | 0.18 (0.02, 0.35) | 0.14 (-0.11, 0.40) | 0.19 (-0.02, 0.41) |
| PFNA | -0.03 (-0.33, 0.28) | -0.41 (-0.86, 0.04) | 0.23 (-0.20, 0.65) |
| ***Central adiposity*** | | | |
| Waist circumference (cm) | | | |
| PFOS | -0.17 (-0.47, 0.14) | -0.28 (-0.72, 0.15) | -0.04 (-0.48, 0.39) |
| PFOA | 0.47 (0.12, 0.82) | 0.98 (0.40, 1.56) | 0.13 (-0.32, 0.58) |
| PFHxS | 0.01 (-0.13, 0.15) | -0.04 (-0.26, 0.18) | 0.03 (-0.15, 0.20) |
| PFNA | -0.09 (-0.35, 0.16) | -0.37 (-0.76, 0.02) | 0.09 (-0.26, 0.43) |
| Waist-to-hip circumference ratio[d] | | | |
| PFOS | -0.24 (-0.72, 0.25) | -0.76 (-1.47, -0.05) | 0.28 (-0.38, 0.93) |
| PFOA | 0.27 (-0.28, 0.82) | 0.84 (-0.09, 1.78) | -0.21 (-0.88, 0.46) |
| PFHxS | -0.03 (-0.25, 0.19) | 0.03 (-0.33, 0.39) | -0.10 (-0.37, 0.17) |
| PFNA | 0.22 (-0.19, 0.63) | 0.40 (-0.23, 1.04) | 0.09 (-0.44, 0.62) |
| Subscapular-to-triceps skinfold thickness ratio[d] | | | |
| PFOS | -0.16 (-1.49, 1.17) | 0.74 (-1.01, 2.49) | -1.21 (-3.26, 0.84) |
| PFOA | 0.58 (-0.94, 2.09) | -0.38 (-2.69, 1.94) | 1.60 (-0.53, 3.72) |
| PFHxS | -0.23 (-0.83, 0.38) | -0.50 (-1.39, 0.39) | 0.01 (-0.84, 0.86) |
| PFNA | 0.46 (-0.68, 1.59) | 0.48 (-1.10, 2.07) | 0.50 (-1.17, 2.17) |

BMI, body mass index; PFAS, perfluoroalkyl substances; PFHxS, perfluorohexane sulfonate; PFNA, perfluorononanoate; PFOA, perfluorooctanoate; PFOS, perfluorooctane sulfonate.

[a]Effect estimates represent the mean change in the outcome for a change in PFAS concentrations from the 25th to the 75th percentile (or interquartile range).

[b]Models adjusted for all four PFAS, maternal age, race/ethnicity, education, parity, pre-pregnancy BMI, timing of sample collection, household income, child sex and age at outcome assessment.

[c]Children with data on BMI and BMI z-score: 988 (522 boys and 466 girls); sum of subscapular and triceps skinfold thickness and subscapular-to-triceps skinfold thickness ratio: 964 (509 boys and 455 girls); waist circumference: 992 (521 boys and 471 girls); and waist-to-hip circumference ratio: 987 (519 boys and 468 girls).

[d]Ratios multiplied by 100 to make estimates interpretable.

**Table S5.** Associations of prenatal plasma PFAS concentrations with overall and central adiposity measurements in mid-childhood among all children and stratified by child sex (models adjusted for all PFAS simultaneously).[a,b,c]

| | All children | Boys | Girls |
|---|---|---|---|
| | **β (95% CI)** | **β (95% CI)** | **β (95% CI)** |
| ***Overall adiposity*** | | | |
| BMI (kg/m$^2$) | | | |
| PFOS | 0.09 (-0.19, 0.36) | 0.02 (-0.37, 0.42) | 0.12 (-0.27, 0.51) |
| PFOA | 0.01 (-0.28, 0.30) | -0.06 (-0.55, 0.45) | 0.03 (-0.33, 0.39) |
| PFHxS | 0.01 (-0.12, 0.14) | -0.07 (-0.29, 0.15) | 0.06 (-0.10, 0.22) |
| PFNA | 0.12 (-0.10, 0.34) | 0.04 (-0.32, 0.40) | 0.22 (-0.06, 0.51) |
| BMI z-score | | | |
| PFOS | 0.06 (-0.03, 0.16) | 0.08 (-0.05, 0.20) | 0.04 (-0.10, 0.17) |
| PFOA | -0.01 (-0.11, 0.09) | -0.11 (-0.27, 0.05) | 0.05 (-0.08, 0.17) |
| PFHxS | -0.01 (-0.05, 0.04) | -0.01 (-0.08, 0.06) | 0.00 (-0.06, 0.05) |
| PFNA | 0.02 (-0.05, 0.09) | 0.02 (-0.10, 0.13) | 0.04 (-0.06, 0.14) |
| Sum of subscapular and triceps skinfold thickness (mm) | | | |
| PFOS | 0.06 (-0.81, 0.94) | 0.19 (-0.97, 1.35) | -0.15 (-1.49, 1.19) |
| PFOA | 0.16 (-0.77, 1.08) | -0.20 (-1.66, 1.26) | 0.42 (-0.84, 1.67) |
| PFHxS | 0.15 (-0.26, 0.56) | -0.28 (-0.91, 0.36) | 0.45 (-0.11, 1.00) |
| PFNA | 0.48 (-0.22, 1.18) | 0.21 (-0.84, 1.27) | 0.81 (-0.17, 1.78) |
| DXA total fat mass index (kg/m$^2$) | | | |
| PFOS | 0.05 (-0.14, 0.24) | 0.04 (-0.22, 0.30) | 0.04 (-0.23, 0.32) |
| PFOA | 0.08 (-0.12, 0.28) | 0.11 (-0.24, 0.46) | 0.08 (-0.17, 0.34) |
| PFHxS | 0.02 (-0.06, 0.11) | -0.06 (-0.20, 0.08) | 0.06 (-0.05, 0.17) |
| PFNA | 0.01 (-0.16, 0.18) | -0.07 (-0.30, 0.16) | 0.12 (-0.14, 0.38) |
| DXA total fat-free mass index (kg/m$^2$) | | | |
| PFOS | 0.07 (-0.06, 0.20) | 0.03 (-0.15, 0.21) | 0.11 (-0.09, 0.30) |
| PFOA | 0.00 (-0.14, 0.14) | -0.02 (-0.26, 0.23) | 0.00 (-0.18, 0.18) |
| PFHxS | -0.01 (-0.07, 0.05) | -0.04 (-0.14, 0.06) | 0.00 (-0.08, 0.08) |
| PFNA | 0.05 (-0.06, 0.17) | 0.09 (-0.07, 0.25) | -0.01 (-0.19, 0.18) |
| ***Central adiposity*** | | | |
| Waist circumference (cm) | | | |
| PFOS | 0.27 (-0.47, 1.00) | 0.14 (-0.87, 1.14) | 0.31 (-0.78, 1.40) |
| PFOA | -0.09 (-0.86, 0.68) | -0.49 (-1.76, 0.77) | 0.09 (-0.92, 1.09) |
| PFHxS | 0.04 (-0.31, 0.38) | -0.15 (-0.70, 0.41) | 0.15 (-0.29, 0.59) |
| PFNA | 0.20 (-0.38, 0.79) | 0.26 (-0.65, 1.18) | 0.29 (-0.49, 1.07) |
| Waist-to-hip circumference ratio[d] | | | |
| PFOS | 0.35 (-0.23, 0.93) | 0.37 (-0.52, 1.27) | 0.23 (-0.50, 0.97) |
| PFOA | -0.31 (-0.91, 0.30) | -0.49 (-1.61, 0.63) | -0.11 (-0.79, 0.56) |
| PFHxS | 0.13 (-0.15, 0.40) | -0.01 (-0.51, 0.49) | 0.19 (-0.10, 0.49) |
| PFNA | 0.27 (-0.20, 0.73) | 0.40 (-0.40, 1.21) | 0.21 (-0.31, 0.74) |
| Subscapular-to-triceps skinfold thickness ratio[d] | | | |
| PFOS | -0.25 (-2.00, 1.49) | 0.48 (-1.90, 2.86) | -1.14 (-3.73, 1.45) |

| | | | |
|---|---|---|---|
| PFOA | 0.23 (-1.62, 2.07) | -1.25 (-4.25, 1.74) | 1.27 (-1.16, 3.69) |
| PFHxS | 0.52 (-0.30, 1.34) | -0.69 (-2.00, 0.61) | 1.43 (0.36, 2.50) |
| PFNA | 1.64 (0.25, 3.04) | 1.69 (-0.47, 3.84) | 1.83 (-0.05, 3.71) |
| DXA trunk fat mass index (kg/$m^2$) | | | |
| PFOS | 0.01 (-0.08, 0.10) | 0.01 (-0.11, 0.12) | 0.01 (-0.12, 0.14) |
| PFOA | 0.05 (-0.04, 0.14) | 0.07 (-0.08, 0.23) | 0.04 (-0.07, 0.16) |
| PFHxS | 0.01 (-0.03, 0.05) | -0.03 (-0.09, 0.04) | 0.03 (-0.02, 0.08) |
| PFNA | 0.01 (-0.06, 0.09) | -0.01 (-0.11, 0.09) | 0.04 (-0.08, 0.16) |

BMI, body mass index; DXA, dual X-ray absorptiometry; PFAS, perfluoroalkyl substances; PFHxS, perfluorohexane sulfonate; PFNA, perfluorononanoate; PFOA, perfluorooctanoate; PFOS, perfluorooctane sulfonate.

[a]Effect estimates represent the mean change in the outcome for a change in PFAS concentrations from the 25th to the 75th percentile (or interquartile range).

[b]Models adjusted for all four PFAS, maternal age, race/ethnicity, education, parity, pre-pregnancy BMI, timing of sample collection, household income, child sex and age at outcome assessment.

[c]Children with data on BMI and BMI z-score: 871 (450 boys and 421 girls); sum of subscapular and triceps skinfold thickness and subscapular-to-triceps skinfold thickness ratio: 873 (451 boys and 422 girls); DXA total fat mass, total fat-free mass, and trunk fat mass indexes: 700 (356 boys and 344 girls); waist circumference: 873 (453 boys and 420 girls); and waist-to-hip circumference ratio: 861 (448 boys and 413 girls).

[d]Ratios multiplied by 100 to make estimates interpretable.

**Table S6.** Associations of prenatal plasma PFAS concentrations with overall and central adiposity measurements in early childhood among all children and stratified by child sex, after adjusting for maternal GFR and albumin levels during pregnancy.[a,b]

| | Additionally adjusted for GFR[c] | | | | Additionally adjusted for albumin[d] | | | |
|---|---|---|---|---|---|---|---|---|
| | All children | Boys | Girls | $p_{int}$ | All children | Boys | Girls | $p_{int}$ |
| | β (95% CI) | β (95% CI) | β (95% CI) | | β (95% CI) | β (95% CI) | β (95% CI) | |
| ***Overall adiposity*** | | | | | | | | |
| BMI (kg/m$^2$) | | | | | | | | |
| PFOS | 0.04 (-0.05, 0.13) | 0.01 (-0.12, 0.15) | 0.05 (-0.07, 0.18) | 0.57 | 0.03 (-0.06, 0.12) | 0.02 (-0.11, 0.15) | 0.02 (-0.10, 0.15) | 0.57 |
| PFOA | 0.09 (-0.02, 0.19) | 0.08 (-0.10, 0.25) | 0.09 (-0.05, 0.23) | 0.83 | 0.08 (-0.03, 0.19) | 0.08 (-0.09, 0.26) | 0.06 (-0.08, 0.20) | 0.81 |
| PFHxS | 0.01 (-0.05, 0.06) | 0.00 (-0.08, 0.08) | 0.01 (-0.06, 0.08) | 0.94 | 0.01 (-0.05, 0.06) | 0.00 (-0.08, 0.08) | 0.00 (-0.07, 0.08) | 0.95 |
| PFNA | 0.03 (-0.07, 0.12) | -0.06 (-0.19, 0.08) | 0.09 (-0.04, 0.22) | 0.20 | 0.02 (-0.07, 0.11) | -0.05 (-0.18, 0.08) | 0.06 (-0.07, 0.19) | 0.20 |
| BMI z-score | | | | | | | | |
| PFOS | 0.03 (-0.03, 0.09) | 0.01 (-0.08, 0.10) | 0.05 (-0.03, 0.13) | 0.50 | 0.03 (-0.03, 0.09) | 0.02 (-0.08, 0.11) | 0.03 (-0.05, 0.12) | 0.51 |
| PFOA | 0.05 (-0.02, 0.12) | 0.02 (-0.10, 0.15) | 0.07 (-0.02, 0.16) | 0.63 | 0.05 (-0.03, 0.12) | 0.03 (-0.09, 0.15) | 0.05 (-0.04, 0.14) | 0.61 |
| PFHxS | 0.01 (-0.03, 0.05) | 0.01 (-0.05, 0.07) | 0.01 (-0.04, 0.06) | 0.86 | 0.01 (-0.03, 0.04) | 0.01 (-0.05, 0.07) | 0.00 (-0.04, 0.05) | 0.85 |
| PFNA | 0.02 (-0.05, 0.08) | -0.04 (-0.13, 0.06) | 0.06 (-0.03, 0.15) | 0.22 | 0.01 (-0.05, 0.08) | -0.03 (-0.13, 0.06) | 0.04 (-0.05, 0.13) | 0.21 |
| Sum of subscapular and triceps skinfold thickness (mm) | | | | | | | | |
| PFOS | -0.08 (-0.34, 0.18) | -0.16 (-0.54, 0.22) | 0.01 (-0.35, 0.37) | 0.48 | -0.11 (-0.37, 0.14) | -0.16 (-0.54, 0.21) | -0.10 (-0.46, 0.27) | 0.45 |
| PFOA | 0.21 (-0.10, 0.52) | 0.43 (-0.07, 0.93) | 0.10 (-0.30, 0.51) | 0.47 | 0.18 (-0.13, 0.49) | 0.42 (-0.07, 0.92) | 0.00 (-0.41, 0.40) | 0.54 |
| PFHxS | 0.17 (0.01, 0.32) | 0.15 (-0.09, 0.39) | 0.19 (-0.02, 0.39) | 0.80 | 0.16 (0.00, 0.31) | 0.15 (-0.09, 0.38) | 0.17 (-0.04, 0.37) | 0.75 |
| PFNA | 0.01 (-0.26, 0.28) | -0.25 (-0.63, 0.14) | 0.24 (-0.14, 0.61) | 0.18 | -0.03 (-0.29, 0.24) | -0.25 (-0.63, 0.14) | 0.13 (-0.25, 0.52) | 0.15 |
| ***Central adiposity*** | | | | | | | | |
| Waist circumference (cm) | | | | | | | | |
| PFOS | 0.04 (-0.18, 0.26) | -0.06 (-0.40, 0.28) | 0.11 (-0.19, 0.41) | 0.57 | 0.05 (-0.17, 0.27) | -0.02 (-0.35, 0.31) | 0.08 (-0.22, 0.38) | 0.61 |
| PFOA | 0.30 (0.03, 0.56) | 0.47 (0.02, 0.91) | 0.17 (-0.16, 0.50) | 0.25 | 0.31 (0.05, 0.57) | 0.51 (0.07, 0.95) | 0.14 (-0.20, 0.47) | 0.24 |
| PFHxS | 0.03 (-0.11, 0.16) | 0.00 (-0.21, 0.21) | 0.04 (-0.13, 0.22) | 0.89 | 0.03 (-0.10, 0.16) | 0.01 (-0.20, 0.22) | 0.04 (-0.13, 0.21) | 0.93 |
| PFNA | -0.02 (-0.25, 0.21) | -0.22 (-0.56, 0.12) | 0.14 (-0.16, 0.45) | 0.35 | -0.01 (-0.24, 0.22) | -0.17 (-0.51, 0.17) | 0.11 (-0.20, 0.42) | 0.37 |
| Waist-to-hip circumference ratio[e] | | | | | | | | |
| PFOS | -0.01 (-0.36, 0.34) | -0.18 (-0.72, 0.37) | 0.13 (-0.32, 0.58) | 0.66 | 0.03 (-0.32, 0.38) | -0.13 (-0.67, 0.41) | 0.12 (-0.33, 0.58) | 0.66 |
| PFOA | 0.17 (-0.25, 0.59) | 0.49 (-0.23, 1.21) | -0.02 (-0.52, 0.48) | 0.13 | 0.21 (-0.21, 0.62) | 0.54 (-0.16, 1.25) | -0.02 (-0.52, 0.48) | 0.12 |

| | | | | | | | | |
|---|---|---|---|---|---|---|---|---|
| PFHxS | -0.01 (-0.22, 0.20) | 0.08 (-0.26, 0.42) | -0.07 (-0.32, 0.19) | 0.44 | 0.00 (-0.21, 0.21) | 0.09 (-0.25, 0.43) | -0.07 (-0.33, 0.19) | 0.44 |
| PFNA | 0.18 (-0.18, 0.54) | 0.34 (-0.21, 0.89) | 0.11 (-0.35, 0.58) | 0.46 | 0.22 (-0.14, 0.58) | 0.39 (-0.15, 0.94) | 0.11 (-0.36, 0.58) | 0.43 |
| Subscapular-to-triceps skinfold thickness ratio[e] | | | | | | | | |
| PFOS | 0.12 (-0.85, 1.08) | 0.47 (-0.87, 1.80) | -0.24 (-1.64, 1.16) | 0.69 | 0.21 (-0.74, 1.17) | 0.54 (-0.77, 1.86) | -0.16 (-1.59, 1.27) | 0.59 |
| PFOA | 0.40 (-0.75, 1.55) | 0.03 (-1.73, 1.78) | 0.61 (-0.96, 2.18) | 0.38 | 0.51 (-0.63, 1.66) | 0.13 (-1.61, 1.87) | 0.78 (-0.79, 2.36) | 0.44 |
| PFHxS | -0.17 (-0.74, 0.41) | -0.39 (-1.22, 0.44) | -0.02 (-0.83, 0.79) | 0.36 | -0.13 (-0.71, 0.44) | -0.34 (-1.17, 0.48) | -0.01 (-0.82, 0.80) | 0.44 |
| PFNA | 0.36 (-0.63, 1.35) | 0.43 (-0.93, 1.78) | 0.19 (-1.28, 1.65) | 0.93 | 0.46 (-0.53, 1.45) | 0.50 (-0.85, 1.85) | 0.33 (-1.16, 1.82) | 0.86 |

BMI, body mass index; GFR, glomerular filtration rate; PFAS, perfluoroalkyl substances; PFHxS, perfluorohexane sulfonate; PFNA, perfluorononanoate; PFOA, perfluorooctanoate; PFOS, perfluorooctane sulfonate.

[a]Effect estimates represent the mean change in the outcome for a change in PFAS concentrations from the 25th to the 75th percentile (or interquartile range).

[b]Children with data on BMI and BMI z-score: 988 (522 boys and 466 girls); sum of subscapular and triceps skinfold thickness and subscapular-to-triceps skinfold thickness ratio: 964 (509 boys and 455 girls); waist circumference: 992 (521 boys and 471 girls); and waist-to-hip circumference ratio: 987 (519 boys and 468 girls).

[c]Models adjusted for maternal age, race/ethnicity, education, parity, pre-pregnancy BMI, timing of blood draw, household income, child sex and age at outcome assessment, and maternal GFR during pregnancy.

[d]Models adjusted for maternal age, race/ethnicity, education, parity, pre-pregnancy BMI, timing of blood draw, household income, child sex and age at outcome assessment, and maternal albumin concentrations during pregnancy.

[e]Ratios multiplied by 100 to make estimates interpretable.

**Table S7.** Associations of prenatal plasma PFAS concentrations with overall and central adiposity measurements in mid-childhood among all children and stratified by child sex, after adjusting for maternal GFR and albumin levels during pregnancy.[a,b]

| | **Additionally adjusted for GFR[c]** | | | | **Additionally adjusted for albumin[d]** | | | |
|---|---|---|---|---|---|---|---|---|
| | **All children** | **Boys** | **Girls** | $p_{int}$ | **All children** | **Boys** | **Girls** | $p_{int}$ |
| | **β (95% CI)** | **β (95% CI)** | **β (95% CI)** | | **β (95% CI)** | **β (95% CI)** | **β (95% CI)** | |
| ***Overall adiposity*** | | | | | | | | |
| BMI (kg/m$^2$) | | | | | | | | |
| PFOS | 0.17 (-0.03, 0.38) | -0.03 (-0.33, 0.28) | 0.30 (0.04, 0.57) | 0.23 | 0.12 (-0.09, 0.32) | -0.03 (-0.33, 0.27) | 0.22 (-0.06, 0.50) | 0.27 |
| PFOA | 0.14 (-0.09, 0.37) | -0.07 (-0.46, 0.33) | 0.24 (-0.03, 0.52) | 0.21 | 0.09 (-0.14, 0.32) | -0.09 (-0.48, 0.31) | 0.17 (-0.10, 0.44) | 0.18 |
| PFHxS | 0.05 (-0.08, 0.17) | -0.07 (-0.27, 0.13) | 0.11 (-0.04, 0.26) | 0.15 | 0.03 (-0.09, 0.16) | -0.07 (-0.27, 0.13) | 0.09 (-0.07, 0.24) | 0.15 |
| PFNA | 0.18 (-0.01, 0.37) | 0.00 (-0.31, 0.30) | 0.33 (0.08, 0.58) | 0.32 | 0.13 (-0.06, 0.33) | -0.02 (-0.33, 0.29) | 0.27 (0.02, 0.52) | 0.28 |
| BMI z-score | | | | | | | | |
| PFOS | 0.08 (0.01, 0.14) | 0.04 (-0.06, 0.14) | 0.10 (0.00, 0.19) | 0.52 | 0.05 (-0.02, 0.12) | 0.03 (-0.07, 0.13) | 0.06 (-0.04, 0.16) | 0.58 |
| PFOA | 0.05 (-0.03, 0.13) | -0.04 (-0.17, 0.08) | 0.10 (0.00, 0.20) | 0.07 | 0.03 (-0.05, 0.10) | -0.05 (-0.18, 0.08) | 0.07 (-0.03, 0.16) | 0.06 |
| PFHxS | 0.01 (-0.03, 0.05) | -0.01 (-0.07, 0.06) | 0.02 (-0.04, 0.07) | 0.55 | 0.00 (-0.04, 0.04) | -0.01 (-0.07, 0.06) | 0.01 (-0.05, 0.06) | 0.54 |
| PFNA | 0.05 (-0.01, 0.12) | 0.02 (-0.08, 0.12) | 0.08 (-0.01, 0.17) | 0.70 | 0.03 (-0.04, 0.09) | 0.01 (-0.09, 0.11) | 0.05 (-0.04, 0.14) | 0.60 |
| Sum of subscapular and triceps skinfold thickness (mm) | | | | | | | | |
| PFOS | 0.55 (-0.09, 1.19) | 0.17 (-0.73, 1.07) | 0.81 (-0.12, 1.74) | 0.41 | 0.33 (-0.32, 0.99) | -0.05 (-0.94, 0.83) | 0.68 (-0.29, 1.65) | 0.45 |
| PFOA | 0.57 (-0.15, 1.29) | -0.02 (-1.18, 1.14) | 0.85 (-0.09, 1.80) | 0.32 | 0.36 (-0.37, 1.08) | -0.33 (-1.50, 0.83) | 0.71 (-0.24, 1.66) | 0.28 |
| PFHxS | 0.27 (-0.12, 0.67) | -0.20 (-0.79, 0.39) | 0.58 (0.05, 1.12) | 0.07 | 0.21 (-0.18, 0.61) | -0.28 (-0.87, 0.30) | 0.55 (0.01, 1.08) | 0.06 |
| PFNA | 0.70 (0.08, 1.31) | 0.22 (-0.68, 1.11) | 1.09 (0.23, 1.95) | 0.44 | 0.50 (-0.12, 1.12) | -0.07 (-0.97, 0.84) | 0.97 (0.10, 1.84) | 0.37 |
| DXA total fat mass index (kg/m$^2$) | | | | | | | | |
| PFOS | 0.14 (-0.01, 0.28) | 0.04 (-0.16, 0.25) | 0.19 (0.00, 0.39) | 0.41 | 0.09 (-0.06, 0.23) | 0.01 (-0.19, 0.21) | 0.14 (-0.06, 0.35) | 0.45 |
| PFOA | 0.16 (0.00, 0.32) | 0.08 (-0.19, 0.36) | 0.19 (0.00, 0.39) | 0.35 | 0.11 (-0.05, 0.27) | 0.04 (-0.24, 0.32) | 0.15 (-0.04, 0.34) | 0.28 |
| PFHxS | 0.05 (-0.03, 0.13) | -0.04 (-0.17, 0.10) | 0.09 (-0.02, 0.20) | 0.11 | 0.04 (-0.05, 0.12) | -0.05 (-0.18, 0.08) | 0.08 (-0.03, 0.19) | 0.10 |
| PFNA | 0.10 (-0.05, 0.25) | -0.02 (-0.21, 0.18) | 0.24 (0.01, 0.47) | 0.27 | 0.05 (-0.10, 0.20) | -0.05 (-0.25, 0.15) | 0.18 (-0.06, 0.41) | 0.29 |
| DXA total fat-free mass index (kg/m$^2$) | | | | | | | | |
| PFOS | 0.08 (-0.01, 0.18) | 0.03 (-0.11, 0.18) | 0.12 (-0.02, 0.25) | 0.64 | 0.09 (-0.01, 0.19) | 0.07 (-0.07, 0.21) | 0.08 (-0.06, 0.23) | 0.61 |
| PFOA | 0.06 (-0.05, 0.17) | 0.02 (-0.18, 0.21) | 0.08 (-0.05, 0.22) | 0.60 | 0.06 (-0.05, 0.17) | 0.07 (-0.12, 0.27) | 0.06 (-0.08, 0.19) | 0.60 |
| PFHxS | 0.00 (-0.05, 0.06) | -0.03 (-0.12, 0.06) | 0.02 (-0.06, 0.09) | 0.52 | 0.00 (-0.05, 0.06) | -0.02 (-0.11, 0.08) | 0.01 (-0.07, 0.08) | 0.52 |
| PFNA | 0.08 (-0.02, 0.19) | 0.07 (-0.06, 0.21) | 0.07 (-0.10, 0.23) | 0.75 | 0.08 (-0.02, 0.19) | 0.12 (-0.02, 0.25) | 0.03 (-0.14, 0.19) | 0.75 |

| | | | | | | | | |
|---|---|---|---|---|---|---|---|---|
| ***Central adiposity*** | | | | | | | | |
| Waist circumference (cm) | | | | | | | | |
| PFOS | 0.41 (-0.12, 0.95) | -0.01 (-0.79, 0.77) | 0.69 (-0.06, 1.43) | 0.35 | 0.21 (-0.33, 0.76) | -0.08 (-0.85, 0.69) | 0.42 (-0.36, 1.20) | 0.41 |
| PFOA | 0.27 (-0.33, 0.88) | -0.32 (-1.33, 0.68) | 0.57 (-0.18, 1.32) | 0.20 | 0.08 (-0.53, 0.68) | -0.45 (-1.46, 0.56) | 0.34 (-0.41, 1.09) | 0.17 |
| PFHxS | 0.12 (-0.20, 0.45) | -0.15 (-0.66, 0.36) | 0.26 (-0.16, 0.69) | 0.24 | 0.07 (-0.26, 0.39) | -0.18 (-0.69, 0.33) | 0.20 (-0.22, 0.63) | 0.23 |
| PFNA | 0.38 (-0.13, 0.89) | 0.13 (-0.65, 0.90) | 0.59 (-0.09, 1.27) | 0.83 | 0.20 (-0.32, 0.71) | 0.01 (-0.77, 0.80) | 0.39 (-0.30, 1.08) | 0.72 |
| Waist-to-hip circumference ratio[e] | | | | | | | | |
| PFOS | 0.41 (-0.02, 0.83) | 0.36 (-0.34, 1.05) | 0.40 (-0.11, 0.90) | 0.66 | 0.39 (-0.04, 0.82) | 0.39 (-0.29, 1.08) | 0.31 (-0.21, 0.84) | 0.70 |
| PFOA | 0.14 (-0.34, 0.61) | 0.00 (-0.89, 0.90) | 0.23 (-0.28, 0.74) | 0.78 | 0.11 (-0.37, 0.59) | 0.07 (-0.83, 0.97) | 0.15 (-0.36, 0.66) | 0.80 |
| PFHxS | 0.19 (-0.07, 0.45) | 0.06 (-0.41, 0.52) | 0.25 (-0.03, 0.54) | 0.75 | 0.19 (-0.07, 0.45) | 0.07 (-0.39, 0.53) | 0.23 (-0.06, 0.52) | 0.74 |
| PFNA | 0.40 (-0.01, 0.80) | 0.42 (-0.26, 1.11) | 0.38 (-0.08, 0.84) | 0.65 | 0.38 (-0.03, 0.79) | 0.50 (-0.20, 1.20) | 0.31 (-0.16, 0.77) | 0.65 |
| Subscapular-to-triceps skinfold thickness ratio[e] | | | | | | | | |
| PFOS | 0.85 (-0.44, 2.13) | 0.40 (-1.45, 2.25) | 1.14 (-0.67, 2.95) | 0.39 | 1.00 (-0.31, 2.31) | 0.32 (-1.51, 2.14) | 1.69 (-0.20, 3.58) | 0.40 |
| PFOA | 0.89 (-0.55, 2.34) | -0.41 (-2.80, 1.98) | 1.45 (-0.38, 3.29) | 0.08 | 1.04 (-0.42, 2.50) | -0.59 (-2.99, 1.82) | 1.86 (0.01, 3.70) | 0.08 |
| PFHxS | 0.71 (-0.08, 1.49) | -0.52 (-1.73, 0.70) | 1.57 (0.54, 2.60) | <0.01 | 0.74 (-0.04, 1.53) | -0.55 (-1.76, 0.66) | 1.68 (0.64, 2.72) | <0.01 |
| PFNA | 1.69 (0.47, 2.92) | 1.23 (-0.61, 3.07) | 2.02 (0.35, 3.69) | 0.42 | 1.85 (0.61, 3.08) | 1.10 (-0.76, 2.96) | 2.40 (0.72, 4.09) | 0.45 |
| DXA trunk fat mass index ($kg/m^2$) | | | | | | | | |
| PFOS | 0.05 (-0.01, 0.12) | 0.02 (-0.07, 0.11) | 0.07 (-0.02, 0.16) | 0.58 | 0.04 (-0.03, 0.10) | 0.01 (-0.08, 0.10) | 0.06 (-0.04, 0.15) | 0.62 |
| PFOA | 0.07 (0.00, 0.14) | 0.06 (-0.07, 0.18) | 0.08 (-0.01, 0.17) | 0.59 | 0.06 (-0.01, 0.13) | 0.05 (-0.08, 0.17) | 0.07 (-0.02, 0.15) | 0.51 |
| PFHxS | 0.02 (-0.02, 0.06) | -0.01 (-0.07, 0.04) | 0.04 (-0.01, 0.09) | 0.14 | 0.02 (-0.02, 0.05) | -0.02 (-0.07, 0.04) | 0.04 (-0.01, 0.08) | 0.13 |
| PFNA | 0.05 (-0.02, 0.11) | 0.01 (-0.08, 0.10) | 0.08 (-0.02, 0.19) | 0.61 | 0.03 (-0.04, 0.10) | 0.00 (-0.09, 0.09) | 0.06 (-0.04, 0.17) | 0.63 |

BMI, body mass index; DXA, dual X-ray absorptiometry; GFR, glomerular filtration rate; PFAS, perfluoroalkyl substances; PFHxS, perfluorohexane sulfonate; PFNA, perfluorononanoate; PFOA, perfluorooctanoate; PFOS, perfluorooctane sulfonate.

[a]Effect estimates represent the mean change in the outcome for a change in PFAS concentrations from the 25th to the 75th percentile (or interquartile range).

[b]Children with data on BMI and BMI z-score: 871 (450 boys and 421 girls); sum of subscapular and triceps skinfold thickness and subscapular-to-triceps skinfold thickness ratio: 873 (451 boys and 422 girls); DXA total fat mass, total fat-free mass, and trunk fat mass indexes: 700 (356 boys and 344 girls); waist circumference: 873 (453 boys and 420 girls); and waist-to-hip circumference ratio: 861 (448 boys and 413 girls).

[c]Models adjusted for maternal age, race/ethnicity, education, parity, pre-pregnancy BMI, timing of blood draw, household income, child sex and age at outcome assessment, and maternal GFR during pregnancy.

[d]Models adjusted for maternal age, race/ethnicity, education, parity, pre-pregnancy BMI, timing of blood draw, household income, child sex and age at outcome assessment, and maternal albumin concentrations during pregnancy.

[e]Ratios multiplied by 100 to make estimates interpretable.

**Table S8.** Longitudinal associations of prenatal plasma PFAS concentrations with overall and central adiposity measurements collected in early and mid-childhood, estimated using linear regression models with generalized estimation equations (GEE).[a,b]

| | All children[c] | Boys[c] | Girls[c] | $p_{int}$ |
|---|---|---|---|---|
| | β (95% CI) | β (95% CI) | β (95% CI) | |
| ***Overall adiposity*** | | | | |
| BMI (kg/m$^2$) | | | | |
| PFOS | 0.10 (-0.02, 0.22) | 0.05 (-0.13, 0.23) | 0.15 (-0.01, 0.31) | 0.35 |
| PFOA | 0.11 (-0.04, -0.25) | 0.04 (-0.20, 0.27) | 0.13 (-0.04, 0.30) | 0.39 |
| PFHxS | 0.02 (-0.06, 0.09) | -0.02 (-0.14, 0.09) | 0.04 (-0.05, 0.13) | 0.41 |
| PFNA | 0.10 (-0.02, 0.22) | -0.03 (-0.21, 0.15) | 0.20 (0.04, 0.36) | 0.16 |
| BMI z-score | | | | |
| PFOS | 0.04 (-0.01, 0.09) | 0.02 (-0.05, 0.10) | 0.05 (-0.02, 0.12) | 0.52 |
| PFOA | 0.04 (-0.02, 0.11) | 0.00 (-0.11, 0.10) | 0.07 (-0.01, 0.15) | 0.19 |
| PFHxS | 0.01 (-0.03, 0.04) | 0.00 (-0.05, 0.05) | 0.01 (-0.03, 0.05) | 0.80 |
| PFNA | 0.03 (-0.03, 0.08) | -0.02 (-0.10, 0.07) | 0.06 (-0.01, 0.13) | 0.25 |
| Sum of subscapular and triceps skinfold thickness (mm) | | | | |
| PFOS | 0.26 (-0.10, 0.62) | 0.13 (-0.39, 0.65) | 0.38 (-0.13, 0.89) | 0.45 |
| PFOA | 0.37 (-0.06, 0.80) | 0.25 (-0.42, 0.93) | 0.40 (-0.15, 0.95) | 0.78 |
| PFHxS | 0.19 (-0.03, 0.42) | 0.04 (-0.29, 0.37) | 0.30 (0.01, 0.60) | 0.31 |
| PFNA | 0.37 (0.00, 0.73) | 0.00 (-0.53, 0.53) | 0.66 (0.15, 1.17) | 0.27 |
| ***Central adiposity*** | | | | |
| Waist circumference (cm) | | | | |
| PFOS | 0.23 (-0.08, 0.54) | 0.07 (-0.39, 0.52) | 0.35 (-0.05, 0.76) | 0.39 |
| PFOA | 0.28 (-0.08, 0.65) | 0.19 (-0.42, 0.79) | 0.29 (-0.15, 0.73) | 0.74 |
| PFHxS | 0.05 (-0.14, 0.24) | -0.04 (-0.33, 0.26) | 0.10 (-0.13, 0.40) | 0.55 |
| PFNA | 0.19 (-0.12, 0.51) | -0.02 (-0.49, 0.45) | 0.37 (-0.04, 0.77) | 0.56 |
| Waist-to-hip circumference ratio | | | | |
| PFOS | 0.20 (-0.08, 0.48) | 0.09 (-0.35, 0.52) | 0.28 (-0.07, 0.63) | 0.96 |
| PFOA | 0.17 (-0.16, 0.50) | 0.30 (-0.28, 0.87) | 0.09 (-0.29, 0.46) | 0.22 |
| PFHxS | 0.07 (-0.10, 0.24) | 0.08 (-0.21, 0.36) | 0.06 (-0.15, 0.26) | 0.73 |
| PFNA | 0.30 (0.02, 0.58) | 0.40 (-0.05, 0.84) | 0.27 (-0.08, 0.61) | 0.46 |
| Subscapular-to-triceps skinfold thickness ratio | | | | |
| PFOS | 0.59 (-0.26, 1.43) | 0.61 (-0.56, 1.78) | 0.53 (-0.69, 1.75) | 0.95 |
| PFOA | 0.66 (-0.33, 1.66) | -0.01 (-1.54, 1.52) | 1.07 (-0.24, 2.39) | 0.14 |
| PFHxS | 0.20 (-0.32, 0.71) | -0.40 (-1.16, 0.35) | 0.65 (-0.06, 1.35) | 0.02 |
| PFNA | 1.15 (0.29, 2.00) | 0.88 (-0.31, 2.08) | 1.38 (0.17, 2.60) | 0.69 |

BMI, body mass index; PFAS, perfluoroalkyl substances; PFHxS, perfluorohexane sulfonate; PFNA, perfluorononanoate; PFOA, perfluorooctanoate; PFOS, perfluorooctane sulfonate.

[a]Effect estimates represent the mean change in the outcome for a change in PFAS concentrations from the 25th to the 75th percentile (or interquartile range).

[b]Models adjusted for maternal age, race/ethnicity, education, parity, pre-pregnancy BMI, timing of blood draw, household income, child sex and age at outcome assessment.

[c]Children with repeated data on BMI and BMI z-score: 1,100 (588 boys and 512 girls); with repeated data on sum of subscapular and triceps skinfold thickness and subscapular-to-triceps skinfold thickness ratio: 1,095 (581 boys and 514 girls); with repeated data on waist circumference: 1,101 (586 boys and 515 girls); and with repeated data on waist-to-hip circumference ratio: 1,097 (585 boys and 512 girls).
